# The Translation and Validation of the Dutch Monash Dog–Owner Relationship Scale (MDORS)

**DOI:** 10.3390/ani9050249

**Published:** 2019-05-16

**Authors:** Emmy A.E. van Houtert, Nienke Endenburg, Joris J. Wijnker, T. Bas Rodenburg, Hein A. van Lith, Eric Vermetten

**Affiliations:** 1 Department of Animal in Science and Society, Utrecht University, 3584 CM Utrecht, The Netherlands; n.endenburg@uu.nl (N.E.); t.b.rodenburg@uu.nl (T.B.R.); H.A.vanLith@uu.nl (H.A.v.L.); 2 Department of IRAS Division EEPI & VPH, Utrecht University, 3548 GM Utrecht, The Netherlands; j.j.wijnker@uu.nl; 3 Department of Psychiatry, Leiden University Medical Centre, 2311 EZ Leiden, The Netherlands; HGJM.Vermetten@mindef.nl; 4 Arq Psychotrauma Expert Groep, 112 XE Diemen, The Netherlands; 5 Department of MGGZ, Ministry of Defence, 3584 EZ Utrecht, The Netherlands

**Keywords:** questionnaire, MDORS, dog, owner, human, relationship, translation, validation

## Abstract

**Simple Summary:**

There are several questionnaires that can evaluate how humans view the relationship they have with their dog. One of those questionnaires is the Monash Dog–Owner Relationship Scale. This questionnaire was originally written for people who speak English. Therefore, it is less useful and also less reliable for people who do not speak English. Since we want the questionnaire to be useful and reliable in more than one language, we wanted to create a reliable translation. The language that we chose for the translation was Dutch. During our translation and reliability study, we found that several of the English questions did not translate well to Dutch. Some words could not be directly translated, and some questions were not interpreted by Dutch-speaking dog owners in the same way that the English questions were interpreted. However, most of the questions were well understood. Therefore, we conclude that a Dutch translation of the Monash Dog–Owner Relationship Scale questionnaire can be used reliably to question Dutch-speaking dog owners after a few adjustments have been made and some questions removed.

**Abstract:**

The Monash Dog–Owner Relationship Scale (MDORS) is a questionnaire that is used to evaluate the perceived relationship between humans and their dog. This questionnaire was originally only formulated and validated in English, which limits its use among non-English speaking individuals. Although a translation could be made, the translation of questionnaires without additional validation often impairs the reliability of that questionnaire. Therefore, the aim of this study was to validate a translation of the MDORS that is suitable for use among native Dutch speakers. To achieve this, a Dutch translation of the MDORS was made and checked for spelling/grammar mistakes, readability, feasibility, and clarity. A test–retest comparison was subsequently performed on the translation together with a calculation of Cronbach’s alpha score and principal component analysis (PCA). Through the PCA, we found that the three-factor model of the original MDORS was also largely present in the Dutch translation. However, deviations were also found, as several questions did not achieve high PCA scores in their original factor. Therefore, we propose that these questions are excluded from the Dutch MDORS.

## 1. Introduction

### 1.1. Describing Human–Animal Relationships

Over the last few decades, various methods have been proposed to objectively and quantitatively evaluate the relationship between humans and companion animals. Examples of such methods include the Pet Attitude Scale [1], the Pet Attachment Survey (PAS) [2], the Companion Animal Bonding Scale [3], the assessment of favorable attitudes toward pets [4], the Lexington Attachment to Pets Scale (LAPS) [5], and the Pet Relationship Scale [6]. Most of these methods are questionnaires that evaluate how a human values their relationship with either pet animals in general, or a specific pet animal. Exactly how these relationships are expressed or described differs between measurement methods and literary sources, as they use different concepts from human psychology to do so. 

The most commonly used concept to quantify human–companion animal relationships seems to be ‘attachment’ [2,5]. Attachment is often used in human psychology to describe a parent–child relationship, and may be defined as a specific aspect of the relationship between parent and child with the purpose of making the child safe, secure, and protected [7]. Since it is originally defined in human–human relationships, it may thus be controversial to use the term ‘attachment’ to describe human–animal relationships. As noted by Ainsworth in 1989 [8], for example, it hadn’t been proven that humans could even be attached to animals, let alone if animals could be attached to either humans or other animals. Studies such as those by Garrity [9], Siegel [10], Johnson et al. [5], and Palmer and Custance [11] have nonetheless used attachment to describe human–animal relationships, while Topal et al. [12] and Prato-Previde et al. [13] tried to clarify the use of the term in their respective articles. Therefore, it remains questionable if humans can be attached to animals in the same way they are to humans, and if attachment is thus a proper way to quantify human–animal relationships.

### 1.2. The Social Exchange Theory

The discussion about whether humans could be attached to companion animals or not was picked up by Dwyer et al. in their article from 2006 [14]. In this article, they discussed human–animal relationships and how these relationships could be quantified. Dwyer et al. [14] noted that the main focus among the quantification of human–animal relationships lay in the expression of the positive effects that these relationships might have on humans [15]. Negative effects remained largely unquestioned, such as the restrictions that pet ownership may put on the social life of pet owners, or the emotional costs of losing a pet [16]. Even if studies questioned the negative effects of pet ownership, they did so without questioning the positive effects, and vice versa. This practice is contrary to the social exchange theory of relationships, which considers any outcome of a relationship between two individuals to be the product of both the costs and benefits for an individual associated with the relationship. Furthermore, it is generally assumed that a relationship is only worthwhile or fulfilling for those involved if the costs and benefits of that relationship are balanced, or if the benefits outweigh the costs [17]. Since it considered both the costs and benefits of relationships, Dwyer et al. [14] proposed using the social exchange theory instead of attachment in the development of a new and more accurate quantification method for human–animal relationships. 

### 1.3. The Monash Dog–Owner Relationship Scale (MDORS)

For their new quantification method, Dwyer et al. [14] chose to evaluate the relationship between humans and dogs. The dog was the first animal to be domesticated by humans. Therefore, it is generally assumed to have the closest relationship with humans out of all animals, which would consequently make their relationship with humans easiest to measure compared to human relationships with other animal species. Dwyer et al. [14] called this quantification the Monash Dog–Owner Relationship Scale. The MDORS was specifically developed to evaluate the relationship between a human and a dog from the human perspective, and to check the strength or impact of the human–animal relationship as experienced by the human. It is a 28-item self-reported questionnaire, which consists of questions divided over three different factors or clusters of related questions: Dog–Owner Interaction (Factor I), Perceived Emotional Closeness (Factor II), and Perceived Costs (Factor III). Answers to all questions can be given on a five-point Likert scale, and the validity of the complete questionnaire was provided by Dwyer et al. in their original publication [14]. 

### 1.4. Validation of Translation

Since its introduction, the MDORS has been used in multiple studies evaluating the relationship between humans and their canine companions. In some of these studies, the MDORS has been translated to other languages, so it would be applicable to a non-native English public. Examples of such translations include German [18], Swedish [19], Danish [20], and Spanish [21]. When translating the MDORS to German, Schoberl et al. [18] investigated how the MDORS behaved among a subject group for which it was not designed (speaking another language and having another culture). They did so by first translating the questionnaire to German in cooperation with a bilingual expert. Next, they presented the translated questionnaire to German-speaking dog owners and performed a principal component analysis (PCA) on the dataset collected from these subjects. Their efforts showed that the translated MDORS did not perform identically to the original English version, as the original three-factor model to group similar questions, as found and proposed by Dwyer et al. [14], did not appear in the German translation. Instead, Schöberl et al. [18] found a five-factor model, which they named: ‘Dog as burden, dog as social supporter, dog as cuddling partner, separation anxiety, and dog as companion’. 

Differences between an original and translated questionnaire are not an uncommon phenomena [18]. Many questionnaire translation processes have found that the translation of a questionnaire from one language to another is not only a translation of words, but also a translation of concepts. This is especially true if one is translating between different cultures and/or ethnicities [22,23,24,25,26]. In their translation of the Satisfaction with Daily Occupation questionnaire, Manee et al. [27] emphasized the importance of culture in translation as they spoke about the influences of cross-cultural adaptation in their translation process. Cross-cultural adaptation is a process that “looks at both language and cultural adaptation issues in the process of preparing a questionnaire for use in another setting’’ [28]. According to this concept, the translation of questionnaires to an audience different than the original is not based solely on textual translation, but requires the re-evaluation of individual questions or groups of questions regarding their distinguishing quality within the questionnaire. Due to cultural differences between populations or differences in textual meaning between languages, translations might lose or change their distinguishing quality and can therefore no longer reliably assess their intended variable. An example of such a loss in value upon translation can be found in the study of Handlin et al. [19] as they translated the MDORS for use among a Swedish population. During their study, Handlin et al. [19] noticed that three questions from their Swedish MDORS translation were interpreted differently by their subject group than intended in the original MDORS. Therefore, these questions no longer measured the concepts for which they were designed, and were eventually removed from the questionnaire to prevent any bias or errors in the scoring that it produced.

### 1.5. Translation Validation for the MDORS

The absence of validation for translated questionnaires is a serious threat to their reliability, accuracy, and comparability to the original. A questionnaire that is translated but not adjusted or re-validated in a new language may not measure the same constructs as its original counterpart, which would mean that it is technically useless in comparative research. Although it has been used in at least five different languages, to our knowledge, only the Swedish and German translations of the MDORS have been checked for deviations from their original counterpart. However, neither has been fully validated on its own, which still makes whether they measure the same constructs as the original or not questionable. Since we want to stress the importance of validating translated questionnaires, and because there is currently no validated MDORS translation available in Dutch, we aim to develop and validate a Dutch translation of the MDORS questionnaire. If a Dutch translation can be validated, the MDORS can in the future not only be used to reliably evaluate the human–animal relationship between dogs and English-speaking individuals, but also between dogs and Dutch-speaking individuals.

## 2. Materials and Methods 

### 2.1. Question Formulation

We used a multi-step process to test the validity and reliability of a Dutch translation of the MDORS questionnaire. This process consisted of the following steps: translation, expert opinion, subject opinion, and statistical analysis. For the translation step, a Dutch native speaker, who was also fluent in English at an academic level and who had worked with the original MDORS, translated the original English questionnaire to Dutch (version T1). They additionally compared their translation to a separate MDORS translation, which had been used in a previous study (version T2) [29]. A Dutch language expert subsequently checked the translations for grammar and spelling mistakes, or strange formulations. Any issues reported during translation or discrepancies between the two translation versions were resolved in this step, resulting in a single translation (version T12). The formulated questions were finally back-translated to English via an automated translation engine. This was done to check for deviations from the original questionnaire based on the factual meaning of each word in the questionnaire, rather than their interpretable meaning to individuals (Table 1). 

### 2.2. Expert and Subject Opinion

After translation, a group of five dog behavior experts who spoke Dutch as their native language and English at an academic level, and were familiar with the original MDORS’s concepts checked the Dutch MDORS for readability (personal opinion), feasibility (missing values <5%), clarity (personal opinion), and discrepancies from the original MDORS (only one of the experts had worked on translations before). The experts additionally judged whether they considered each individual question essential to the questionnaire. From the answer to this latter question, a ‘Content Validity ratio’ (CVR) [30] was calculated for each individual question. This was done via the following formula.

CVR = [(E − (N/2))/(N/2)].(1)

In this formula, E stands for the number of experts in the panel that thought the question was essential to the questionnaire, while N stands for the total number of experts in the panel. Calculated scores could range between 1 (very essential) and –1 (absolutely not essential).

A group of test subjects additionally judged the questions for readability, feasibility, and clarity, in addition to filling out the questionnaire. These individuals were recruited by spreading a recruitment message on various social media platforms. A total of 501 individuals responded to the recruitment message and answered the questionnaire. This group had an average age of 39.62 years (±13.63), a 95% female to 5% male ratio, and lived distributed across the Netherlands. 

### 2.3. Statistical Analysis

A sample of 88 subjects (of the original 501) were willing to fill out the questionnaire a second time to help test for test–retest reliability via a Pearson coefficient. The intermediate period between both inquiries was two weeks. This period was chosen based on earlier test–retest protocols for questionnaires by Brazier et al. [31]. 

The construct validity of the Dutch MDORS was tested with a principal component analysis (PCA) [32]. We calculated the PCA of the Dutch MDORS according to the three-factor model that was found by Dwyer et al. [14] (see Table 2 for question division). Through the use of PCA, clusters of related questions could be identified within the questionnaire. This was done by comparing the calculated PCA score of all the questions to one another for score height and orientation (positive or negative). Scores of individual questions had to be above 0.4 or below −0.4 (maximum score between −1 and 1) to consider a question relevant for a specific cluster. Furthermore, the percentage-explained variance of the total variance was calculated for each identified question cluster. The explained variance of a question cluster had to be above 5% for the questions within that cluster to be considered relevant to the overall outcome of the questionnaire. 

A Cronbach’s alpha score was finally calculated for each of the three factors originally found by Dwyer et al. [14] to determine if the internal consistency of the Dutch MDORS differed from the internal consistency found for the factors in the original MDORS [33]. The advisable cut-off point used for Cronbach’s alpha was 0.7 or above for good internal consistency [34,35]. 

## 3. Results

### 3.1. Question Formulation

We encountered no major issues during the translation process of the English MDORS. Only five disputable points were identified. These points concerned questions number 3, 9, 19, 20, and 28. In question number 3, the translation of the words ‘food treats’ was difficult, as its literal translation ‘voedseltraktaties’ is not commonly used in the Dutch language to describe rewards for dogs. However, no other suitable Dutch translation could be found without the risk of losing the original meaning of the words. Therefore, we chose to use the literal translation in the questionnaire. A similar problem was encountered with question number 9, as an adequate translation for the word ‘groom’ could not be found in Dutch. Therefore, we decided to describe the Dutch term instead of translating the word. The resulting replacement was: ‘het verzorgen van de vacht van de hond’ (to take care of your dog’s coat). After receiving advice from a Dutch language expert, we did not use the word ‘traumatisch’ (traumatic) in question number 19. This word is not commonly used in daily Dutch language, which could cause the misinterpretation of questions in which it is used. Instead, the word ‘moeilijk’ (difficult) was used. In question number 20, the word ‘chore’ was difficult to translate directly to Dutch due to several translation options also being able to mean other words. The word ‘karwei’ was eventually chosen, although this was mistranslated to the word ‘job’ upon back-translation. This is not a common Dutch interpretation of the word, as job is usually translated as either ‘baan’ or ‘werk’. Finally, in question number 28, the back-translations differed from the original English MDORS in context. This is due to the limitation of automated translation, because it can only provide literal translations and cannot fully capture contextual or cultural differences in languages. The product of the translation process can be found in Table 1.

### 3.2. Expert and Subject Opinion

The expert group considered all the questions proposed for the Dutch translation of the MDORS essential for the questionnaire (CVR = 1.0). The readability (0 issues reported), feasibility (missing values 0%), and clarity (0 issues reported) of all the questions was additionally deemed sufficient by both the expert and subject group.

### 3.3. Pearson Coefficient

To test for test–retest reliability, a Pearson coefficient was calculated over the MDORS scores of the 88 subjects who filled out the questionnaire twice. This was done by separately calculating the MDORS score each of the two times the participants filled out the questionnaire. Then, the scores of the first were compared to the scores of the second, which yielded a Pearson coefficient of 0.83. 

### 3.4. PCA

Through the PCA (Table 2), we found a three-factor model among the Dutch MDORS, which resembled the model found by Dwyer et al. [14] in their original validation of the MDORS. The questions originally belonging to Factor I in the English MDORS were also found in a single question cluster in our translation, with the exception of questions 3 and 8. The questions of Factor I are further all oriented in the same direction.

The questions that study the emotional closeness of owners to their dog (Factor II) are also mostly found in a single question cluster. All but two (questions 18 and 19) had high (over 0.6) component scores, which were additionally oriented in the same direction. 

The questions that study the perceived cost of dog ownership (Factor III) are also mainly found in a single question cluster. The only exception is question 25, though this is only due to its component score being just below the acceptable level of 0.4 (score 0.37). Similar to the other two question clusters, all the questions are again oriented in the same direction.

### 3.5. Cronbach’s Alpha

We calculated Cronbach’s alpha of the Dutch MDORS translation for each of the three factors described by Dwyer et al. [14] in the original MDORS. The found scores were 0.43 for Factor 1, 0.19 for Factor 2, and 0.19 for Factor 3 (compared to respectively 0.67, 0.84, and 0.84 in the original MDORS). According to the accepted interpretation of Cronbach’s alpha [34,35], these scores indicated poor internal consistency for all of the factors of the questionnaire. 

## 4. Discussion

### 4.1. Statistical Considerations

The conception of the Dutch MDORS was not without some statistical or design issues. A first issue was that some concepts or sentences could not be literally translated to the Dutch language. Therefore, the adjustment of some questions was necessary to make the MDORS applicable to a Dutch population. Cronbach’s alpha for all three factors was additionally calculated to be below 0.7 (0.43, 0.19, and 0.19), which would suggest poor internal consistency. Although we do not have a definite explanation for the low Cronbach’s alpha scores that were found for the Dutch MDORS, we do know that it was not caused by low response numbers, as is sometimes encountered in questionnaires (N = 501) [36]. Therefore, the most likely reason for the change in alpha that we can devise from the literature on the topic is that the Cronbach’s alpha score might be low in our translation due to several items measuring heterogeneous constructs or related questions that have lost their explanatory value in translation due to cultural differences between dog owners. This theory is supported the results found in the PCA and the relatively high (0.83) Pearson coefficient, which measures internal consistency independent of inter-item relatedness [36]. The Cronbach’s alpha score might additionally be low due to the low number of items in the test (28). Fewer items can lead to an alpha score that is artificially low because it cannot accurately estimate the items’ relation to the measured construct [36]. However, this is a less likely explanation, as the alpha scores of the original MDORS were high, and also appear to have been calculated over a total of 28 items.

Finally, it should be noted that female subjects were overrepresented in this study’s subject group (95% female versus 5% male). This is a known phenomenon in voluntary questionnaires [19,21,37,38,39,40]. A possible way to equalize the male–female ratio among subjects would be to actively ask men to participate in the questionnaire. However, this practice could lead to differences in subject motivation, or may introduce a selection bias because the researcher has to actively choose and approach subjects. Therefore, we decided that it wasn’t desirable to actively attempt to equalize the male–female ratio among respondents during this study. However, this leads to the possibility that the results of this study are not representative for male dog owners. 

### 4.2. Similarity between the Original and Translated MDORS

Besides some statistical considerations, the Dutch MDORS translation seems to fit the results that were found by Dwyer et al. [14] for the original English version of the questionnaire. The three factors or question clusters found by Dwyer et al. [14] can also clearly be identified in our Dutch translation. These factors are: Dog–Owner Interaction (Factor I), Perceived Emotional Closeness (Factor II), and Perceived Costs (Factor III). Still, some questions did not seem to fit the original MDORS model, as the PCA showed them to score below the cut-off point of 0.4. In Factor I, this concerned questions 3 and 8. In Factor II, this concerned questions 18 and 19, while in Factor III, it concerned question 25. 

Although it is not entirely clear why these questions did not relate fully to the other questions in their original factor, some explanations can be proposed. In Factor I, it should be noted that questions 3 and 8 scored only slightly below the cut-off point (0.39 and 0.36, respectively). Some other questions of Factor I additionally only scored slightly above the cut-off point (between 0.40–0.45). Therefore, it could be possible that instead of only questions 3 and 8, all the questions belonging to Factor I together are not that related to the rest of the MDORS, and some are only included because of the placement of the cut-off point. This is supported by the percentage of explained variation in the questionnaire associated with Factor I, as it is only 9.58% of the total variation.

Regarding Factor II, it appears that question 18 is associated more with the questions placed in Factor I than its original Factor II. This might be because of the concept measured by the question, as it asks owners how often they share secrets with their dog that they do not share with other people. The use of the term often implies frequency, which is perhaps associated more with Factor I as it measures frequency of interaction, rather than Factor II, which measures emotional attachment. Question 19 is also more closely associated with the questions of Factor I than Factor II, although the reasoning behind this association is similarly uncertain. Similar to question 18, the association might be due to the concept addressed by the question, as it asks owners how difficult it would be for them if their dog died. The death of their dog would prevent owners from performing any interactive behaviors with it; therefore it is more closely associated to questions of interaction frequency. 

Finally, regarding Factor III, it appears that question 25 scores just below the cut-off point (0.4) for any association with this factor. This might be due to the possibility of an open interpretation of this question. The possibility for the open interpretation of this question lies in the use of the word messy, as the word’s exact interpretation may differ between individuals. The downside of open interpretation in MDORS questions has already been studied by Handlin et al. [19] in their Swedish MDORS translation. In their translation, they found that questions 15 and 23 could be interpreted differently in Swedish than intended by the original authors of the MDORS. After consideration, they eliminated these questions from the Swedish MDORS, because a change in interpretation would influence the scoring system that can be applied to the MDORS.

### 4.3. The Impact of Phenotype

Besides the exclusion of questions based on our statistical results, we would also like to address two non-statistical points of discussion detected in the original MDORS. The first point is the possibility of bias in the MDORS. In their work from 1996, Zasloff et al. [41] stated that the quantification methods of human–animal relationships may be biased due to the way in which their questions are formulated. They specifically addressed interspecies bias, as some interaction behaviors that are used to measure the strength of human–animal relationships may be observed more in one species than in another due to species-specific behavior. Therefore, the assessment of a human–animal relationship based on these interaction elements could create the impression that relationships with an animal that can perform the behavior are stronger than with an animal that cannot perform the behavior. However, this may not be true, as different animal species, or even individual animals, have different ways of expressing themselves. 

In their original MDORS, Dwyer et al. [14] aimed to avoid the issue of species bias by designing their questionnaire to be applicable only to dogs. However, since they chose the dog we question if Dwyer et al. [14] did not indivertibly encounter another type of bias in their questionnaire: phenotypical bias. Due to the high phenotypical variation among dogs, some phenotypes may look even less related than two different animal species. Although behavior patterns generally do not differ strongly between dogs, that they look very different from one another may influence the way they interact with humans in a similar manner as species-specific behavior. Good examples of this possibility are questions 2 and 9 of the original MDORS. Question 2 asks how often people take their dog with them when they visit other people, while question 9 asks how often people groom their dog. These questions may not be answered based solely on the basis of the dog–owner relationship, as they may be affected by the size and breed of the dog. For example, short-haired dogs require less grooming than long-haired dogs, while small dogs are easier to carry everywhere than large ones. However, this does not necessarily mean that owners of short-haired or large dogs interact less with their dog or that they do not have a close emotional relationship with their animal. Therefore, we advise that all questions that are related to the transport or grooming of dogs are removed from all versions of the MDORS to prevent a bias of phenotype on the evaluation of human–animal relationships via the questionnaire. 

In fact, we advise that no question belonging to Factor I should be counted toward the overall MDORS given how much discussion is possible regarding some of these questions. For example, Handlin et al. [19] also found in their study that not all dog owners were able to answer question 5 of Factor I (How often do you take you dog in the car?), since not all owners owned a car. Instead, we propose that the questions of Factor I are instead used as an indicator of how likely the outcomes of factors II and III are to be true for the owner who filled out the questionnaire, as they do indicate how often dogs and owners are in direct contact with one another.

### 4.4. Are Costs and Benefits Balanced in the MDORS?

The second point that we would like to discuss regarding both the original and Dutch MDORS is the use of the social exchange theory. In their original publication, Dwyer et al. [14] stated that the MDORS was based on social exchange theory [17]. This is apparent in the inclusion of both positive and negative aspects of the human–dog relationship in the MDORS. However, we question if the positive and negative aspects of the human–dog relationship are measured equally in the MDORS. According to social exchange theory, it is generally assumed that a relationship is only worthwhile or fulfilling for those involved if the costs and benefits of that relationship are balanced, or if the benefits outweigh the costs. Despite it being based on this concept, it remains to be seen if the balance between costs and benefits can indeed be measured by the MDORS. Within the MDORS, only one positive aspect (emotional attachment) of the human–animal relationship is measured, while several negative aspects are measured (pain of loss, financial impact, impact on daily life). Therefore, one could argue that the measured positive and negative aspects are not in balance, because several potential positive aspects of dog ownership go unquestioned. Examples include the connection between some dog breeds and human social status [42], the higher chance of social interaction while being with a dog [43], or the dog providing meaning and structure to the life of their owner. Therefore, it would be advisable to investigate if the addition of more questions regarding the emotional benefits of dog ownership could be a meaningful addition to the existing questions of the MDORS. 

## 5. Conclusions

In conclusion, we found that a translated MDORS can be used to assess dog owner relationships as perceived by the owner among a population of Dutch native-speaking dog owners. However, we advise that questions 18, 19, and 25 of the original MDORS are not used in the Dutch version based on their low PCA scores. Questions 2, 5, and 9 should additionally be removed from Factor I, although the argument to do so is based on logical reasoning rather than statistical analysis. Based on logical reasoning, it would further be advisable to remove the remaining questions of Factor I entirely from the MDORS scale calculation. Questions regarding the positive effects of human–animal relationships may be added to the scoring system instead in order to balance the number of questioned costs and benefits of human–animal interaction, although the further study of these questions is required. The questionnaire resulting from all of these proposed changes can be found in Appendix A Table A1.

## Figures and Tables

**Table 1:** The original English version of the Monash Dog–Owner Relationship Scale (MDORS) by Dwyer et al. [14] compared to the Dutch native speaker translation, and the automated back-translation. Deviations from the original MDORS are marked in **Bold** in the back-translation.

Original English MDORS Version (Dwyer et al., 2006)	Dutch Translation	Literal Automated Back-Translation
1. How often do you play games with your dog?	Hoe vaak speelt u met uw hond?	How often do you play with your dog?
2. How often do you take your dog to visit people?	Hoe vaak neemt u uw hond mee op visite?	How often do you take your dog **with you on a visit?**
3. How often do you give your dog food treats?	Hoe vaak geeft u uw hond voedsel traktaties?	How often do you give your dog food treats?
4. How often do you kiss your dog?	Hoe vaak geeft u uw hond een kusje?	How often do you **give your dog a kiss?**
5. How often do you take your dog in the car?	Hoe vaak neemt u uw hond mee in de auto?	How often do you take your dog in the car?
6. How often do you hug your dog?	Hoe vaak knuffelt u uw hond?	How often do you **cuddle** your dog?
7. How often do you buy your dog presents?	Hoe vaak koopt u “cadeautjes” voor uw hond?	How often do you buy **“presents” for your dog?**
8. How often do you have your dog with you while relaxing, i.e., watching TV?	Hoe vaak is uw hond bij u terwijl u ontspant, (bijv.TV kijken)?	How often is your dog with you while you **are relaxing (e.g., watching TV)?**
9. How often do you groom your dog?	Hoe vaak verzorgt u de vacht van uw hond?	How often do you **take care of your dog’s coat?**
10. My dog helps me get through tough times.	Mijn hond helpt me door moeilijke tijden.	My dog helps **me through** difficult times.
11. My dog is there whenever I need to be comforted.	Mijn hond is er voor me wanneer ik getroost moet worden.	My dog is there **for me when I have** to be comforted.
12. I would like to have my dog near me all the time.	Ik zou mijn hond graag altijd bij me hebben.	**I would always like to have my dog with me.**
13. My dog provides me with constant companionship.	Mijn hond biedt me altijd gezelschap.	**My dog always offers me company.**
14. If everyone else left me, my dog would still be there for me.	Als iedereen me zou verlaten, zou mijn hond er nog voor me zijn.	If **everyone left** me, my dog would still be there for me.
15. My dog gives me a reason to get up in the morning.	Mijn hond geeft me een reden om ‘s ochtends op te staan.	My dog gives me a reason to get up in the morning.
16. I wish my dog and I never had to be apart.	Ik zou willen dat mijn hond en ik nooit uit elkaar hoefden te zijn	I wish my dog and I never had to be **separated.**
17. My dog is constantly attentive to me.	Mijn hond heeft altijd aandacht voor mij.	My dog **always has attention for** me.
18. How often do you tell your dog things you don’t tell anyone else?	Hoe vaak vertelt u uw hond dingen die u aan niemand anders vertelt?	How often do you tell your dog things **that you tell no one** else?
19. How traumatic do you think it will be for you when your dog dies?	Hoe moeilijk zou het voor u zijn als uw hond overleed?	How **difficult would it be for you if your dog died?**
20. How often do you feel that looking after your dog is a chore?	Hoe vaak voelt het verzorgen van uw hond als een karwei?	How often **does taking care of your dog feel like a job?**
21. It is annoying that I sometimes have to change my plans because of my dog.	Het is vervelend dat ik soms mijn plannen moet veranderen vanwege mijn hond	It is annoying that **sometimes I** have to change my plans because of my dog.
22. It bothers me that my dog stops me doing things I enjoyed doing before I owned it.	Het is vervelend dat mijn hond me weerhoudt van dingen die ik deed voordat ik hem/haar had	It **is annoying** that my dog **keeps** me from doing things **that I did before I had him/her.**
23. There are major aspects of owning a dog I don’t like.	Er zijn belangrijke aspecten aan het bezitten van een hond die ik niet leuk vind	There are **important** aspects of owning a dog that I do not like.
24. How often does your dog stop you doing things you want to?	Hoe vaak weerhoudt uw hond u ervan dingen te doen die u wilt doen?	How often does your dog **prevent you from** doing things that you want to do?
25. My dog makes too much mess.	Mijn hond maakt te veel rommel.	My dog makes too much mess.
26. My dog costs too much money.	Mijn hond kost te veel geld.	My dog costs too much money.
27. How hard is it to look after your dog?	Hoe moeilijk is het om voor uw hond te zorgen?	How **difficult** is it **to take care of** your dog?
28. How often do you feel that having a dog is more trouble than it is worth?	Hoe vaak heeft u het gevoel dat het hebben van een hond meer kost dan het oplevert?	How often do you feel that having a dog **costs more than it produces**?

**Table 2:** The results of the principal component analysis (PCA) of the Dutch translation of the MDORS questionnaire (N = 501). The analysis was performed according to the three factors originally found by Dwyer et al. [14], which resulted in the production of three score clusters. Component scores above 0.4 or below −0.4 are marked in **bold**.

		Cluster[1]	Cluster[2]	Cluster[3]
Factor Questions	% of Variance	19.87	10.04	9.58
1. How often do you play games with your dog?		0.07	0.06	**0.55**
2. How often do you take your dog to visit people?		−0.07	0.05	**0.41**
3. How often do you give your dog food treats?		0.02	−0.10	0.39
4. How often do you kiss your dog?		0.10	0.06	**0.55**
5. How often do you take you dog in the car?		−0.03	−0.08	**0.42**
6. How often do you hug your dog?		0.05	−0.06	**0.43**
7. How often do you buy your dog presents?		0.13	0.01	**0.56**
8. How often do you have your dog with you while relaxing, i.e., watching TV?		0.12	−0.03	0.36
9. How often do you groom your dog?		0.03	0.18	**0.45**
10. My dog helps me get through tough times.		**0.87**	0.01	0.14
11. My dog is there whenever I need to be comforted.		**0.86**	0.03	0.07
12. I would like to have my dog near me all the time.		**0.78**	0.09	0.26
13. My dog provides me with constant companionship.		**0.90**	−0.00	0.01
14. If everyone else left me my dog would still be there for me.		**0.90**	−0.00	0.01
15. My dog gives me a reason to get up in the morning.		**0.64**	0.01	0.26
16. I wish my dog and I never had to be apart.		**0.73**	0.11	0.28
17. My dog is constantly attentive to me.		**0.73**	0.15	−0.00
18. How often do you tell your dog things you don’t tell anyone else?		0.14	0.08	**0.55**
19. How traumatic do you think it will be for you when your dog dies?		−0.15	−0.00	**−0.43**
20. How often do you feel that looking after your dog is a chore?		0.00	**0.60**	−0.02
21. It is annoying that I sometimes have to change my plans because of my dog.		0.23	**0.68**	0.06
22. It bothers me that my dog stops me doing things I enjoyed doing before I owned it.		0.28	**0.66**	0.00
23. There are major aspects of owning a dog I don’t like.		0.33	**0.61**	0.07
24. How often does your dog stop you doing things you want to?		−0.06	**0.56**	−0.05
25. My dog makes too much mess.		−0.09	0.37	0.06
26. My dog costs too much money.		−0.11	**0.48**	0.08
27. How hard is it to look after your dog?		0.10	**0.48**	0.00
28. How often do you feel that having a dog is more trouble than it is worth?		0.00	**0.41**	−0.09

## Appendix A

**Table A1:** The questions that were retained in the Dutch MDORS.

	Minstens 1 Keer Per Dag	2 tot 3 Keer per Week	1 Keer per Week	Minstens 1 Keer per Maand	(Bijna) Nooit
Hoe vaak speelt u met uw hond?	O	O	O	O	O
Hoe vaak geeft u uw hond voedsel traktaties?	O	O	O	O	O
Hoe vaak geeft u uw hond een kusje?	O	O	O	O	O
Hoe vaak knuffelt u uw hond?	O	O	O	O	O
Hoe vaak koopt u “cadeautjes” voor uw hond?	O	O	O	O	O
Hoe vaak is uw hond bij u terwijl u ontspant, (bijvoorbeeld tijdens televisie kijken)?	O	O	O	O	O
	Heel erg mee oneens	Mee oneens	Neutraal	Mee eens	Heel erg mee eens
Mijn hond helpt me door moeilijke tijden.	O	O	O	O	O
Mijn hond is er voor me wanneer ik getroost moet worden.	O	O	O	O	O
Ik zou mijn hond graag altijd bij me hebben.	O	O	O	O	O
Mijn hond biedt me altijd gezelschap.	O	O	O	O	O
Als iedereen me zou verlaten, zou mijn hond er nog voor me zijn.	O	O	O	O	O
Mijn hond geeft me een reden om ’s ochtends op te staan.	O	O	O	O	O
Ik zou willen dat mijn hond en ik nooit uit elkaar hoefden te zijn.	O	O	O	O	O
Mijn hond heeft altijd aandacht voor mij.	O	O	O	O	O
Het is vervelend dat ik soms mijn plannen moet veranderen vanwege mijn hond	O	O	O	O	O
Het is vervelend dat mijn hond me weerhoudt van dingen die ik deed voordat ik hem/haar had.	O	O	O	O	O
Er zijn belangrijke aspecten aan het bezitten van een hond die ik niet leuk vind	O	O	O	O	O
Mijn hond kost te veel geld.	O	O	O	O	O
	Minstens 1 keer per dag	2 tot 3 keer per week	1 keer per week	Minstens 1 keer per maand	(Bijna) nooit
Hoe vaak heeft u het gevoel dat het hebben van een hond meer kost dan het oplevert?	O	O	O	O	O
Hoe vaak voelt het verzorgen van de hond als een karwei?	O	O	O	O	O
Hoe vaak weerhoudt uw hond u ervan dingen te doen die u wilt doen?	O	O	O	O	O
	Heel erg moeilijk	Een beetje moeilijk	Neutraal	Niet moeilijk	Helemaal niet moeilijk
Hoe moeilijk is het om voor uw hond te zorgen?	O	O	O	O	O
